# Enhanced Responsiveness to Selective Serotonin Reuptake Inhibitors during Lactation

**DOI:** 10.1371/journal.pone.0117339

**Published:** 2015-02-17

**Authors:** Nicholas J. Jury, Betsy A. McCormick, Nelson D. Horseman, Stephen C. Benoit, Karen A. Gregerson

**Affiliations:** 1 Neuroscience Graduate Program, College of Medicine, University of Cincinnati, Cincinnati, Ohio, 45267, United States of America; 2 Division of Pharmaceutical Sciences, James L. Winkle College of Pharmacy, University of Cincinnati, Cincinnati, Ohio, 45267, United States of America; 3 Department of Molecular and Cellular Physiology, University of Cincinnati, Cincinnati, Ohio, 45267, United States of America; 4 Division of Reproductive Endocrinology and Infertility, Department of Obstetrics and Gynecology, University of Cincinnati, Cincinnati, Ohio, 45267, United States of America; 5 Department of Psychiatry, College of Medicine, University of Cincinnati, Cincinnati, Ohio, United States of America; University of Rennes-1, FRANCE

## Abstract

The physiology of mood regulation in the postpartum is poorly understood despite the fact that postpartum depression (PPD) is a common pathology. Serotonergic mechanisms and their dysfunction are widely presumed to be involved, which has led us to investigate whether lactation induces changes in central or peripheral serotonin (5-HT) systems and related affective behaviors. Brain sections from lactating (day 10 postpartum) and age-matched nulliparous (non-pregnant) C57BL/6J mice were processed for 5-HT immunohistochemistry. The total number of 5-HT immunostained cells and optical density were measured. Lactating mice exhibited lower immunoreactive 5-HT and intensity in the dorsal raphe nucleus when compared with nulliparous controls. Serum 5-HT was quantified from lactating and nulliparous mice using radioimmunoassay. Serum 5-HT concentrations were higher in lactating mice than in nulliparous controls. Affective behavior was assessed in lactating and non-lactating females ten days postpartum, as well as in nulliparous controls using the forced swim test (FST) and marble burying task (MBT). Animals were treated for the preceding five days with a selective serotonin reuptake inhibitor (SSRI, citalopram, 5mg/kg/day) or vehicle. Lactating mice exhibited a lower baseline immobility time during the FST and buried fewer marbles during the MBT as compared to nulliparous controls. Citalopram treatment changed these behaviors in lactating mice with further reductions in immobility during the FST and decreased marble burying. In contrast, the same regimen of citalopram treatment had no effect on these behaviors in either non-lactating postpartum or nulliparous females. Our findings demonstrate changes in both central and peripheral 5-HT systems associated with lactation, independent of pregnancy. They also demonstrate a significant interaction of lactation and responsiveness to SSRI treatment, which has important implications in the treatment of PPD. Although recent evidence has cast doubt on the effectiveness of SSRIs, these results support their therapeutic use in the treatment of PPD.

## Introduction

Mood alterations during the postpartum and postpartum depression (PPD) adversely affect not only the mother, but also disrupt bonding and the health of the child [1]. The relationship between untreated maternal depression and negative infant outcomes, even through adolescence, are well established [2,3,4]. PPD (defined in the psychiatric nomenclature as a major depressive disorder with a specifier of onset during pregnancy and/or following childbirth) affects 10–20% of women who give birth [5,6,7,8].

From a biological perspective, it is an evolutionary imperative that female mammals cope with the physiological stresses of pregnancy, child birth, and lactation without suffering the debilitations inherent with PPD. From this biological perspective, attention naturally focuses on PPD as a disorder, and several studies have suggested specific mechanisms of PPD [9;10,11].

The control of mood and the etiology of depressive disorders in particular, are not completely understood. However, substantial evidence has accrued that serotonergic systems play a central role [1,12,13,14]. Genetic variants in components of the serotonergic system have been correlated with depression [15]. Altered function of the serotonin transporter (SERT) or tryptophan hydroxylase (TPH) has been found in PPD subjects [1,14,15]. Levels of serotonin (5-HT) and its major metabolite, 5-hydroxyindoleacetic acid (5-HIAA), are significantly lower in the cerebrospinal fluid of depressed patients and in brain tissue of suicide victims [16,17,18]. Reduced availability of the 5-HT precursor, tryptophan, has also been found in depressed patients [19]. Moreover, SSRIs are the first line of pharmacotherapy in PPD and relieve depressive symptoms in most of these patients [4,20].

Although there is evidence that SSRIs are effective in treating PPD [4,21,22], there is still much debate about the effectiveness of SSRIs in treating depressive disorders. Two independent research consortiums conducted meta-analyses on clinical trials submitted to the Food and Drug Administration and determined that the therapeutic effect of the SSRIs were relatively small when compared to placebo in severely depressed patients [23,24]. In contrast, two other independent research teams conducted meta-analyses and concluded that SSRIs were effective in treating depressive symptoms when compared to placebo regardless of the severity of the depressive symptoms [25,26].

In 2004 a novel serotonergic biosynthetic system in the mammary gland was identified and found to be highly upregulated during late pregnancy and lactation [27]. This discovery provides a new context in which to consider whether serotonergic systems are altered in the postpartum, and ultimately whether the central and peripheral serotonergic systems influence one another during this time. This study presents our initial examination of these serotonin systems in the context of the lactating animal, using a selective SSRI (citalopram) with which to probe the behavioral responsiveness of the central serotonin system. Here we investigated the biochemical changes in central (dorsal raphe nucleus) and peripheral (serum) 5-HT systems in lactating mice using immunohistochemistry and radioimmunoassay, respectively. We also examined the effect(s) of sub-chronic SSRI treatment on affective state, as measured by depression-related (forced swim test, FST) and anxiety-related behaviors (marble burying task, MBT) in the postpartum. The present studies compared these behaviors among normal lactating and non-lactating dams without experimentally induced depression. This experimental design was chosen in lieu of a model of maternal depression in order to examine the effects of lactation on the serotonergic systems and affective behavior during a period of peak lactation, day 10 postpartum [27,28,29]. Herein the data demonstrate an enhanced responsiveness to SSRI treatment in lactating dams compared with non-lactating females. We also report changes in both central and peripheral 5-HT systems during lactation. The culmination of these results have clinical implications in the treatment of PPD.

## Materials and Methods

### Subjects

**Fig 1:**
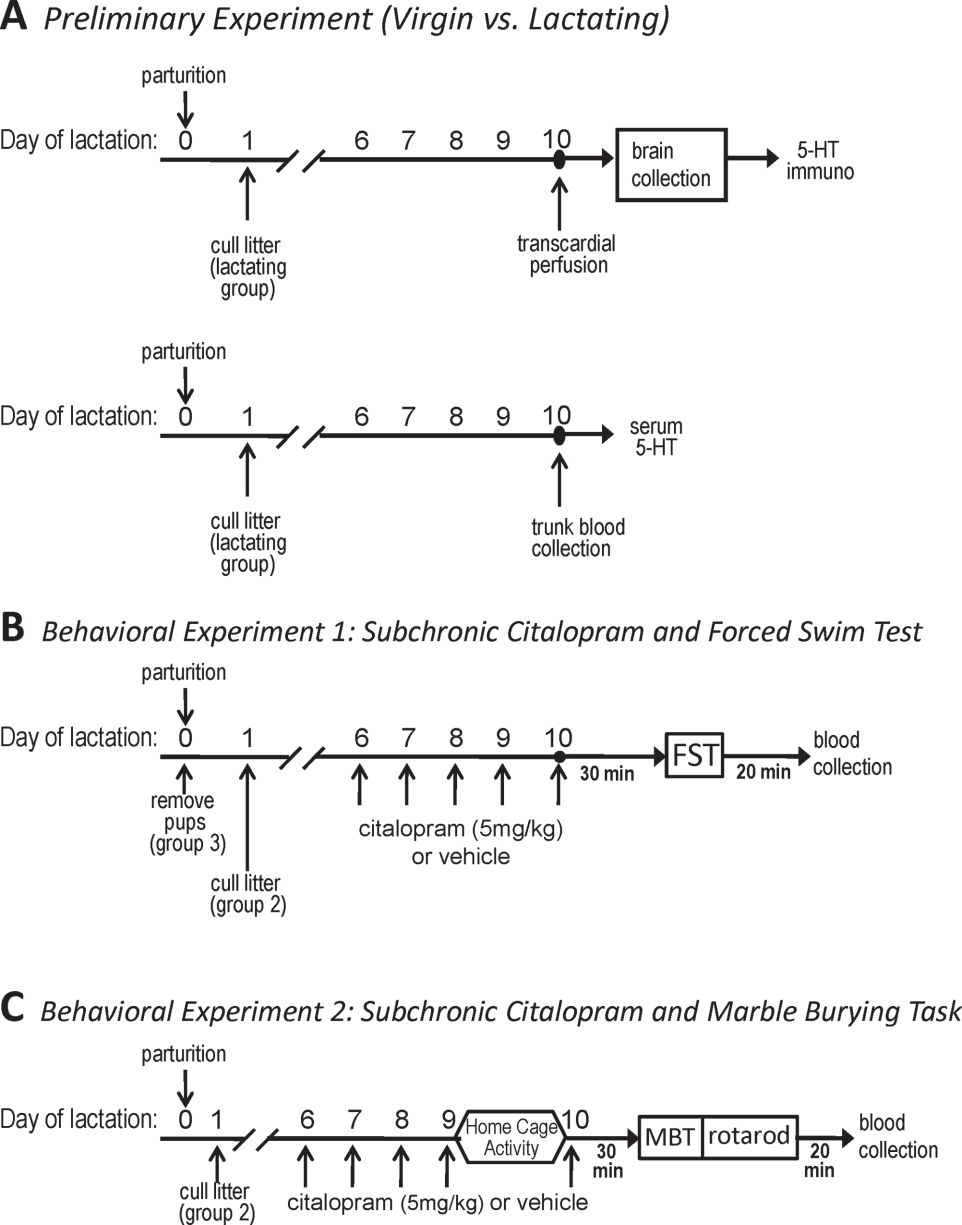
Schemas for experiments assessing behavior after multiple days of citalopram treatment. ***A***: Preliminary Experiment (Virgin vs. Lactating): 1) *Top panel*: virgin and lactating (day 10 postpartum) mice were perfused and brain tissue was collected for immunohistochemical analysis of 5-HT. 2) *Bottom panel*: virgin and lactating (day 10 postpartum) mice were terminated and trunk blood was collected for determination of serum 5-HT via radioimmunoassay. ***B***: Behavioral Experiment 1 in which three groups were studied: 1) nulliparous females; 2) lactating mice; and 3) postpartum-nonlactating mice. Only the FST was administered in this study. ***C***: Behavioral Experiment 2 examined only two groups: 1) nulliparous females and 2) lactating mice. This study included “control” behavioral tests (home cage activity and motor performance task) and an alternate test of mood-related behavioral assessment—the marble burying test (MBT)

C57BL/6J mice (age 12–20 weeks) were used in these studies. The initial analysis of immunoreactive 5-HT was performed on brains from lactating dams (day 10 postpartum; Fig. 1A, top panel, *see below*) and age-matched virgin females. Virgin and lactating (day 10 postpartum) mice were also used for the initial analysis of serum 5-HT (Fig. 1A, bottom panel). For subsequent studies involving behavioral analyses, all females were housed with a stud male for two weeks, checked for vaginal plugs, then separated and housed individually [30,31] on a 12h:12h light:dark cycle with water and standard lab chow available *ad libitum*. Mated mice that did not produce a litter were assigned to the “nulliparous”, non-pregnant groups. The breeding pairs yielded a mating success rate of 47%, which is consistent with other literature reporting the mating behavior of the C57BL/6J inbred mouse line [32]. Mice that became pregnant and delivered pups had their litters culled to six pups on day 1 postpartum (day of parturition = day 0) to normalize for suckling stimulus, and these constituted the “lactating” groups. In behavioral experiment 1(see Fig. 1B), a third group of dams was included (“postpartum-nonlactating”), which were mice that had their entire litters removed immediately after delivery (postpartum day 0). All procedures were reviewed and approved by the University of Cincinnati’s Institution for Animal Care and Use Committee.

### Experimental Designs

**Preliminary Experiment: Dorsal Raphe 5-HT Immunohistochemistry Virgin vs. Lactating Mice.** Female C57BL/6J mice ∼12–16 weeks in age (n = 3 virgin, n = 4 lactating) were used for the initial immunohistochemical analyses for 5-HT. Animals were deeply anesthetized with an i.p. injection of ketamine-xylazine (90mg/kg and10mg/kg, respectively) 10 minutes prior to the perfusion procedure (Fig. 1A, top panel). Animals underwent transcardial perfusion with 10mL of 0.01M phosphate buffered saline (PBS) followed by 20mL of 4% paraformaldehyde. Brains were removed (Fig. 1A, top panel), post-fixed overnight in 4% PFA, and then stored in 30% sucrose at 4°C until they sank. Brains were sectioned on a freezing microtome at 30 μm coronal slices between -4.84 Bregma and -5.02 Bregma, according to a mouse brain stereotaxic atlas [33].

Brain slices were immunostained for 5-HT as free-floating sections, after which they were mounted on slides. Briefly, sections were rinsed with ice-cold 0.01 M PBS (5 min, 3x), then incubated for 60 minutes in blocking buffer (0.01 M PBS containing 3% rabbit serum, 2% BSA, and 0.4% Triton X-100), followed by additional washes (0.01M PBS; 5 min, 3x). Sections were then incubated for 16 hours (room temp) with rat monoclonal anti-5-HT (1:200 in 0.01 M PBS + 0.4% Triton X-100). Following rinses in ice-cold 0.01 M PBS (5 min, 3x), sections were incubated for 60 min (room temp) with biotinylated rabbit anti-rat IgG (1:5000 in 0.01 M PBS + 0.4% Triton X-100). Sections were then processed according to manufacturer’s instructions, using the Vectastain *Elite* ABC immunoperoxidase system and Ag/Ab complexes were visualized with Ni^2+^-DAB enzyme substrate. Brain slices from virgin and lactating mice were exposed to the Ni^2+^-DAB solution for identical periods of time (5 min; in the same solution). Negative controls omitted the primary antibody from the 16-hr incubation.

Stained brain sections were analyzed using the open source NIH Image software on a Macintosh computer. The border of the dorsal raphe was defined [33] and background determined on neighboring tissue. In the thresholding mode (background subtracted) the number of stained cell bodies within the dorsal raphe and their optical densities were documented for each section. Rat polyclonal anti-5-HT and biotinylated rabbit anti-rat antibodies were purchased from Chemicon International (Billerica, MA) and Vectastain *Elite* ABC immunoperoxidase system from Vector Laboratories Ltd (Burlingame, CA).

**Preliminary Experiment: Serum 5-HT Blood Collection and Radioimmunoassay Virgin vs. Lactating Mice.** Trunk blood samples were collected from virgin (n = 30) and lactating (n = 24, day 10 postpartum) mice ∼12–20 weeks of age (Fig. 1A, bottom panel). Blood samples were allowed to clot overnight at 4°C to release 5-HT from platelets. Serum was separated by centrifugation at 12,000 rpm for 15 minutes at 4°C, and stored at -80°C until assayed. 5-HT levels were determined in duplicate using a commercial RIA kit according to the manufacturer’s instructions. The sensitivity of the RIA kit for serum 5-HT was 6.7 ng/mL and the interassay coefficient of variation was less than 5.0%. 5-HT RIA Fast Track kits were purchased from Rocky Mountain Diagnostics (Colorado Springs, CO). Citalopram was purchased from Tocris Biosciences (Ellisville, MO). Fluoxetine and all other chemicals, unless otherwise noted, were purchased from Sigma-Aldrich (St. Louis, MO).

**Forced Swim Test Pilot Experiment.** Citalopram dose and duration used in the experiments described below were based on published literature, which has used male mice almost exclusively [34,35]. To confirm the reproducibility of the published results and the FST technique by our laboratory, a preliminary study was conducted to confirm the efficacy of this sub-chronic SSRI treatment (*S1A Fig.)*. Male mice (age ∼12–16 weeks, n = 15 per group) received daily i.p. injections of citalopram, (5mg/kg/day) or vehicle (0.9% saline) between 08:00h and 09:30h for 5 days. Each animal was subjected to the FST thirty minutes after the final injection.

**Behavioral Experiment 1: Subchronic Citalopram and Forced Swim Test.** Using the FST as an assay of depressive-like behavior, three groups of female mice were tested: Group 1, nulliparous (non-pregnant, age ∼12–18 weeks; n = 30 vehicle, n = 17 citalopram); Group 2, lactating (age ∼12–18 weeks; n = 20 vehicle, n = 18 citalopram); and Group 3, postpartum-nonlactating (age ∼12–18 weeks; n = 7 vehicle; n = 7 citalopram; *see* Fig. 1B). On day 6 postpartum, dams of Groups 2 and 3, and age-matched nulliparous females (Group 1) began receiving daily i.p. injections of SSRI (citalopram, 5mg/kg/day) or vehicle (0.9% saline). Injections were administered between 08:00h and 09:30h for 5 days. Thirty minutes after the fifth injection on day 10 postpartum, a period of peak lactation [27,28,29], each animal was subjected to the FST.

**Behavioral Experiment 2: Subchronic Citalopram and Marble Burying Task.** The effect of SSRI treatment was tested again, using the marble burying task (MBT), an assay of anxiety-like behavior. In addition, two other behaviors (home cage activity and motor performance) were analyzed to determine if differences were specific to affective behavior. Only lactating dams (age ∼12–16 weeks; n = 8 per group) and age-matched nulliparous females (n = 10 per group) were used in this study (*see* Fig. 1C), and all animals received daily i.p. injections of citalopram (5mg/kg/day) or vehicle for five days. As in behavioral experiment 1 (Fig. 1B), injections in the lactating dams began on day 6 postpartum. Between the fourth and fifth injection, home cage activity was monitored for 24 hours (see Fig. 1C). Thirty minutes after the last injection, each animal was assessed with the MBT and then tested for motor performance using a rotarod (see Fig. 1C).

**Behavioral Experiment 3: Increased Citalopram Dosage and Longer Administration.** Nulliparous female mice did not exhibit any changes in FST behavior following a five day treatment with citalopram (5mg/kg/day). In light of these results we decided to increase the dose of citalopram from 5 mg/kg to 30 mg/kg, and from five days of treatment to ten days in nulliparous mice (age ∼15 weeks; n = 6 per group) prior to the FST (see *S1B Fig.*).

**Behavioral Experiment 4: Acute Fluoxetine and Forced Swim Test.** In order to rule out a citalopram-specific effect in the nulliparous mice, we decided to treat with fluoxetine, another SSRI with different pharmacokinetics [50]. Lactating mice (day 10 postpartum, age ∼16 weeks; n = 14 vehicle, n = 10 (10mg/kg), n = 9 (40mg/kg)) were treated with a single dose of either fluoxetine (10mg/kg or 40mg/kg), or vehicle 30 minutes prior to the FST (see *S1C Fig., left panel)*. The higher dose (40mg/kg) was found to be effective in reducing immobility during the FST in lactating mice (see *S3A Fig.*). Nulliparous mice (age ∼ 12–14 weeks; n = 11 per group) were given a single injection of fluoxetine (40mg/kg) or vehicle 30 minutes prior to the FST (see *S1C Fig., right panel*).

### Behavioral Tests

Mice were removed from the housing area and taken to a separate room for all behavioral testing. Mice were allowed to acclimate to the testing room for approximately one hour.

**Forced Swim Test.** The FST was always administered between 09:00h and 11:30h. Mice were placed in a swim tank (height = 30cm, diameter = 20cm) containing water (25°C ± 1°C) at a height of 20cm for a total of six minutes. All sessions were recorded on video and scored by two independent observers who were blinded to the treatments. The total time of immobility was recorded. The first two minutes were not used in the determination of the total immobility time as per Porsolt et al. [36]. A mouse was deemed to be immobile when it was floating and making only minor movements to keep its head above water. Tanks were emptied and rinsed clean after each animal.

**Home Cage Activity.** In behavioral experiment 2 (see Fig. 1C), locomotor activity within the home cage was monitored for 24 hours beginning 30 minutes after the penultimate injection of vehicle or citalopram. The home cage was placed in a SmartFrame stainless steel rack (Hamilton-Kinder Scientific Company, Poway, CA) and infrared photobeam interruption recorded both vertical and horizontal movements. Data were collected and analyzed using HMM100 MotorMonitor software and the total numbers of basic movements were broken into 60-minute time intervals.

**Marble Burying Task.** For the marble burying task (MBT) in behavioral experiment 2 (see Fig. 1C), each test mouse was placed into a larger, novel cage with a 5cm layer of sawdust on top of the bedding. In the lactating group, each dam was transferred to the novel cage without their pups (the home cages containing the pups were placed into a separate room so the ultrasonic vocalizations of the pups did not influence the behavioral testing). Mice were allowed to acclimate in the novel cage for 30 minutes after which 20 clean, transparent glass marbles (1.5 cm diameter) were placed on top of the sawdust, in five rows of four marbles each, with the marbles spaced equally apart. At the end of 20 minutes, the number of buried marbles (at least two-thirds covered with sawdust) was recorded [37,38]. This design was chosen following a preliminary experiment in which the effects of novel cage versus separation from pups on marble burying behavior in lactating dams was assessed (see *S4 Fig.*). In that experiment, marbles were either introduced into the home cage (pups remaining with some dams and removed from others), or in a novel cage setting (some dams having been transferred with their pups and others without their pups).

**Motor Performance Task.** Motor performance was determined using a rotarod (see Fig. 1C). Mice were placed on a stationary drum (∼62 mm diameter) that then began to revolve at a constant speed. The total amount of time the mouse remained on the rotarod was recorded during each of three trials administered 30 minutes apart. The first (acclimation) trial used a speed of 16 rpm for 120 seconds, or until the mouse failed to remain on the revolving drum. The second and third trials used a speed of 20 rpm for up to 300 seconds [39].

### Blood Collection and Assays

**Serum 5-HT: Measurement following FST and MBT.** 20 minutes after the FST (Behavioral Experiment 1; Fig. 1B) or rotarod test (Behavioral Experiment 2; Fig. 1C), animals were terminated and trunk blood collected. The blood samples were allowed to clot overnight at 4°C to release serotonin (5-HT) from platelets. Serum was separated by centrifugation at 12,000 rpm for 15 minutes at 4°C, and stored at -80°C until assayed. 5-HT levels were determined in duplicate using a commercial RIA kit according to the manufacturer’s instructions. The sensitivity of the RIA kit for serum 5-HT was 6.7 ng/mL and the interassay coefficient of variation was less than 5.0%. 5-HT RIA Fast Track kits were purchased from Rocky Mountain Diagnostics (Colorado Springs, CO). Citalopram was purchased from Tocris Biosciences (Ellisville, MO). Fluoxetine and all other chemicals, unless otherwise noted, were purchased from Sigma-Aldrich (St. Louis, MO).

### Data Analysis

Data obtained in each experiment were analyzed using either one-way or two-way analysis of variance (ANOVA) followed by Bonferonni post hoc test or Dunn’s post hoc test for comparison of the means. In some experiments when only two means were compared, a two-tailed Student’s t-test (unpaired) was used. All data shown are presented as the mean ± standard error of the mean (SEM). All experiments were designed to achieve a statistical power of 80 percent. Means were considered to be significantly different when *p*<0.05. All statistical tests were performed using Graph Pad Prism statistical software.

## Results

### Preliminary Experiment: Dorsal Raphe 5-HT Immunohistochemistry Virgin vs. Lactating Mice

**Fig 2:**
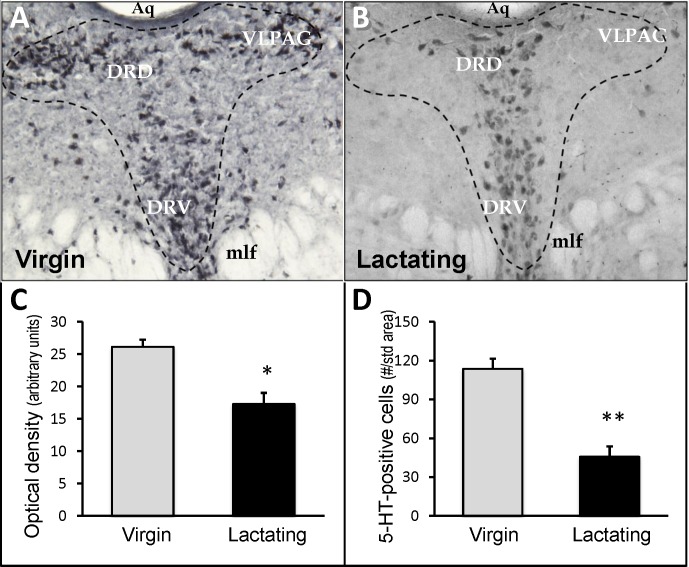
Serotonin immunostaining of the dorsal raphe nucleus (DRN) immediately caudal to oculomotor nucleus. **(Bregma -4.48 mm)**. Representative examples of sections processed for 5-HT immunostaining and visualized with Ni^2+^-DAB (blue-black stain) from virgin female mice (*A*; n = 3) and lactating mice (*B*; n = 4). *C*: Average optical density of cell bodies (background-subtracted; threshold detection) in the field as demarcated by the dashed lines in *A* and *B. D*: Average number of detectable stained cells in the field. *Aq*, Aqueduct of Sylvius; *DRD*, dorsal raphe-dorsal part; *DRV*, dorsal raphe-ventral part; VLPAG, ventrolateral aspect of the periaqueductal gray; mlf, medial longitudinal fasciculus. Student’s t-test comparison of the means: *p<0.05, **p<0.01 compared to virgin controls.

In all sections of the dorsal raphe, immunoreactive 5-HT was noticeably less in the lactating mice as compared to virgin mice, examples of which are presented in Fig. 2, A and B. The differences in 5-HT immunoreactivity were particularly evident in the lateral “wings” of the dorsal raphe nucleus (DRN)—the ventrolateral aspect of the periaqueductal gray (VLPAG). Image analysis of the staining indicated that both the number of stained cells and the intensity (over background) of staining were significantly reduced in the DRN of lactating mice (Fig. 2, C and D [t = 12.48, df = 6, *p*<0.01]).

### Preliminary Experiment: Serum 5-HT Virgin vs. Lactating Mice

**Fig 3:**
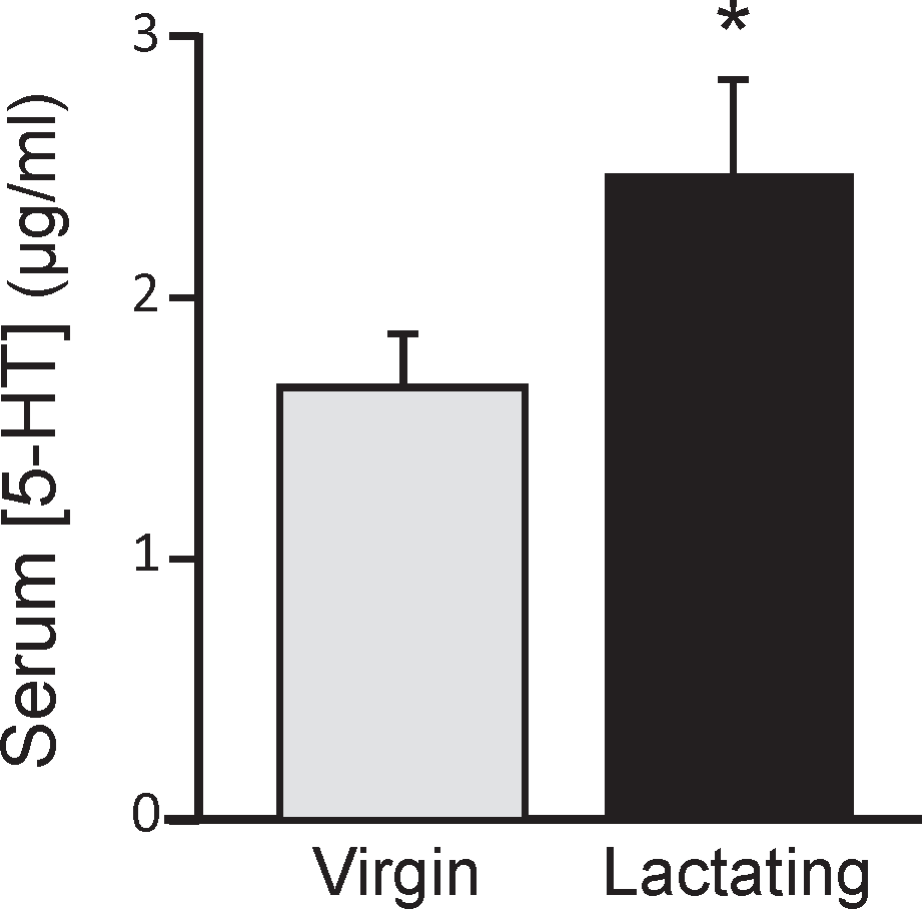
Serum levels of serotonin in virgin female and lactating C57Bl/6J mice. Trunk blood was allowed to clot overnight, releasing platelet 5-HT stores. Lactating mice had significantly higher levels of serum 5-HT than virgin females. Student’s t-test comparison of the means: *p<0.05 compared to virgin controls (n = 24–30 per group).

Serum 5-HT was significantly elevated in lactating mice compared with virgin female mice (Fig. 3 [t = 2.220, df = 52, *p*<0.05]). Nearly all circulating 5-HT is sequestered in the dense granules of the platelets and is released into the serum upon clotting [40].

### Forced Swim Test Pilot Experiment

Male mice treated with citalopram via injection for five days (5mg/kg/day), showed a significant [(t = 2.550, df = 28, *p*<0.05)] reduction in immobility time in the FST as compared to vehicle-treated controls (*S2A Fig.*), as has been widely reported in the literature [34,35].

### Behavioral Experiment 1: Subchronic Citalopram and Forced Swim Test

**Fig 4:**
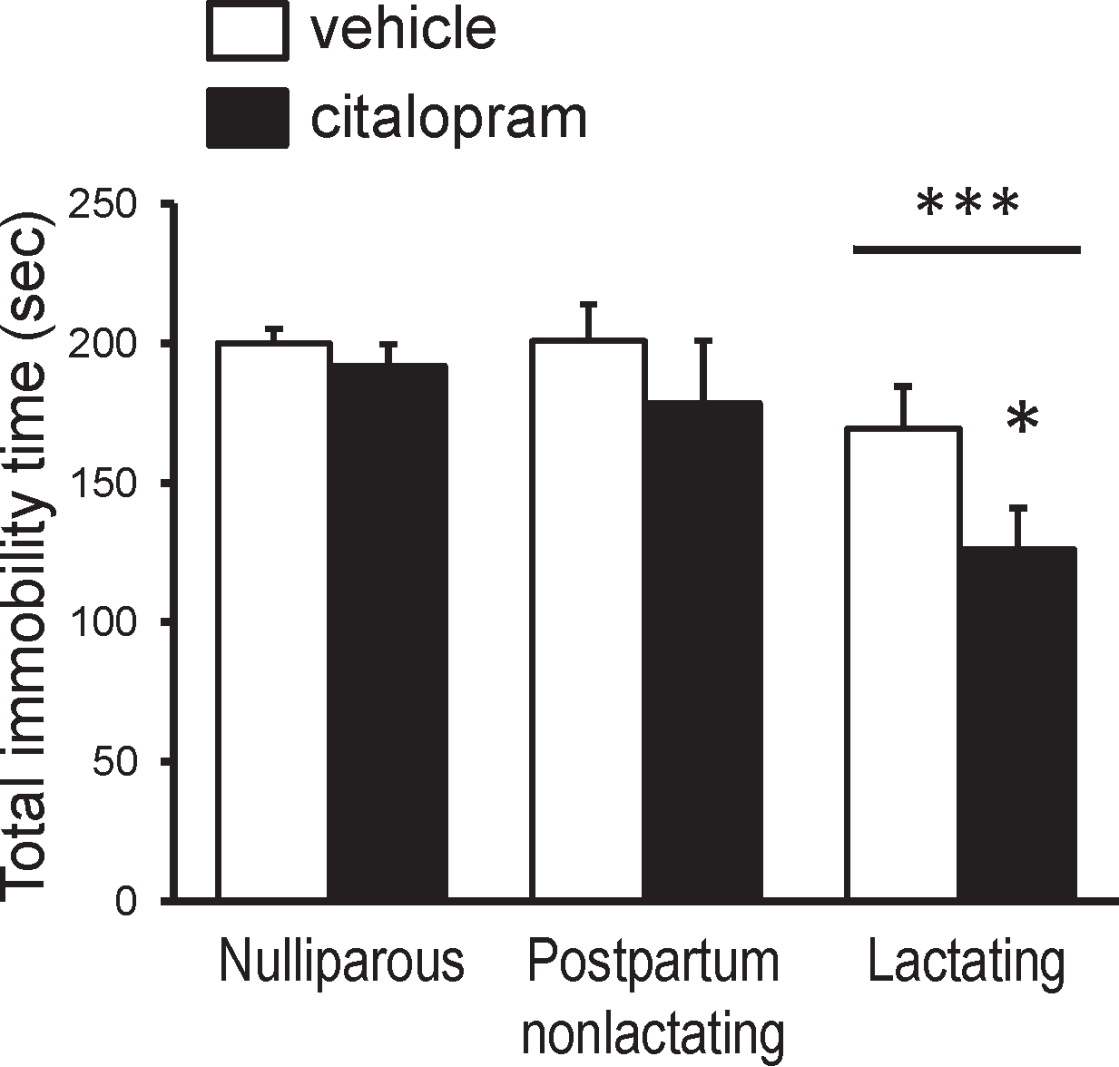
In the FST, lactating mice respond to regimen of citalopram treatment that has no effect on nulliparous or postpartum-nonlactating female mice. Mice received daily injections of vehicle or citalopram (5mg/kg/day, i.p.) for 5 days. Total immobility time (excluding the first two minutes of the test). Bonferonni post hoc test comparison of the means: *p<0.05 compared to vehicle-treated lactating mice; ***p<0.001 overall effect of lactation determined by 2-way ANOVA (n = 7–30 per group).

In female mice subjected to the FST there was a significant main effect of lactation state (Fig. 4), such that the lactating females overall had lower immobility times than either of the non-lactating groups as determined by two-way ANOVA [F(2,93) = 11.23, *p*<0.001]. There was also a main effect of drug treatment [F(1,93) = 5.033, *p* = 0.0272] on immobility during the FST, but there was no interaction between lactation and drug treatment. In response to citalopram the lactating females showed a significant response to the SSRI as demonstrated by a decrease in immobility time [(t = 2.820, df = 36, *p*<0.05, compared with vehicle-treated)]. However, neither of the non-lactating groups (nulliparous or pup-deprived) responded to citalopram (Fig. 4).

### Behavioral Experiment 2: Subchronic Citalopram and Marble Burying Task

To verify the differential responsiveness of lactating and non-lactating mice to SSRIs, and also to determine if these responses are specific to affective behavior, multiple behaviors were assessed following five days of citalopram treatment (*see* Fig. 1C).

### Marble Burying Task

**Fig 5:**
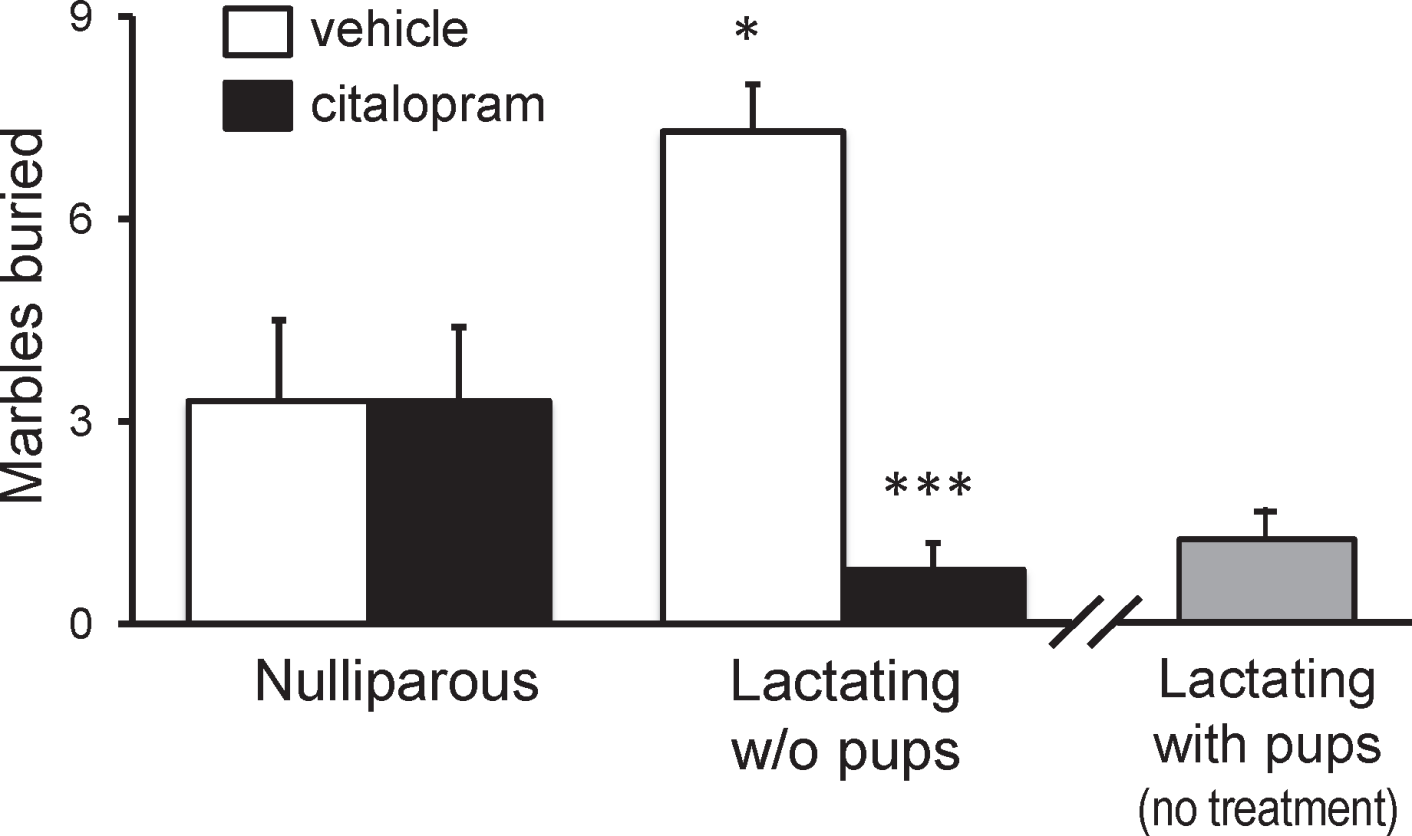
In the marble-burying test, lactating mice respond to citalopram treatment that has no effect on nulliparous mice. All mice were moved to a novel cage for testing. Lactating mice were tested while separated from their pups. Student’s t-test comparisons of the mean: *p<0.05 compared to vehicle-treated nulliparous mice; ***p<0.001 compared to vehicle-treated lactating group (n = 5–10 per group).

Affective behavior was assessed using the MBT, a measurement of anxiety-like behavior. Separation of dams from their pups during the MBT caused elevated anxiety-like behavior (more marbles buried) [41,42,43], which was independent of whether they remained in the home cage, or were moved to a novel cage (*S4 Fig.*). Lactating mice kept with their pups buried so few marbles (1.2 ± 0.7, with several dams burying no marbles at all) that an effect of SSRI treatment would be impossible to measure. Thus, we chose to test citalopram on dams moved to a novel cage without their pups (Fig. 5). Similar to results in the FST, non-lactating females exhibited no behavioral changes in the MBT following citalopram treatment (five days, 5mg/kg/day). Two-way ANOVA revealed an interaction between lactation and drug treatment on marble burying behavior [F(1,32) = 13.11, *p* = 0.0010, Fig. 5]. Lactating females experienced a significant reduction in anxiety-like behavior in response to citalopram (Fig. 5 [t = 4.857, df = 16, p<0.0001]). Notably, the marble-burying activity of citalopram-treated lactating females in the absence of their pups was similar to that in mothers with their pups present (Fig. 5).

### Home Cage Activity

**Fig 6:**
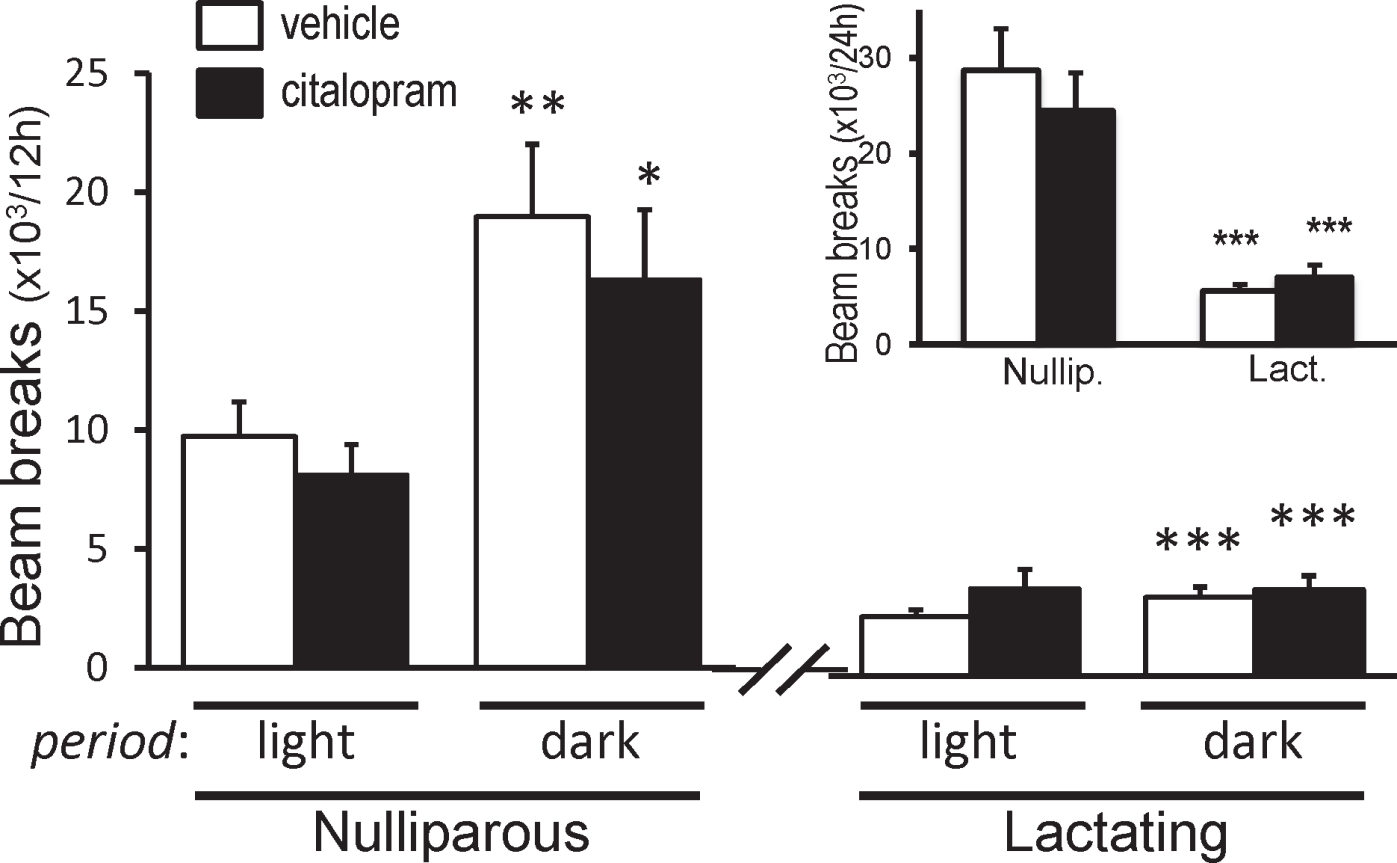
Citalopram has no effect on home cage activity of either nulliparous or lactating mice. Activity was monitored over a 24-h period and data is shown for light and dark periods separately or for entire 24-h period (*Inset*). Citalopram treatment did not affect home cage activity in any of the groups. Note significant increase in activity of nulliparous animals during the dark period. Bonferonni post hoc test for comparison of the means: *p<0.05, **p<0.01 activity in dark vs. corresponding light period. 2-way ANOVA***p<0.001 lactating vs. corresponding nulliparous group (n = 8–10 per group).

SSRI treatment did not significantly alter locomotor activity measured during the 24 hour period between the 4^th^ and 5^th^ injection of citalopram in either lactating or nulliparous mice. Not surprisingly, lactating dams had significantly less overall home-cage activity than nulliparous mice (Fig. 6, *inset* [F(1,64) = 64.4; *p*<0.0001]). Since rodents are nocturnal animals, the home cage activity was divided into light and dark phases and reanalyzed. SSRI treatment did not alter locomotor activity in either lactating or nulliparous controls during the light or dark phases of the cycle (Fig. 6). As expected, nulliparous animals exhibited an increase in locomotor activity during the dark phase. However, lactating dams (day 9–10 postpartum) did not exhibit any significant changes in locomotor activity between the dark and light phases (Fig. 6).

### Motor Performance Task

**Fig 7:**
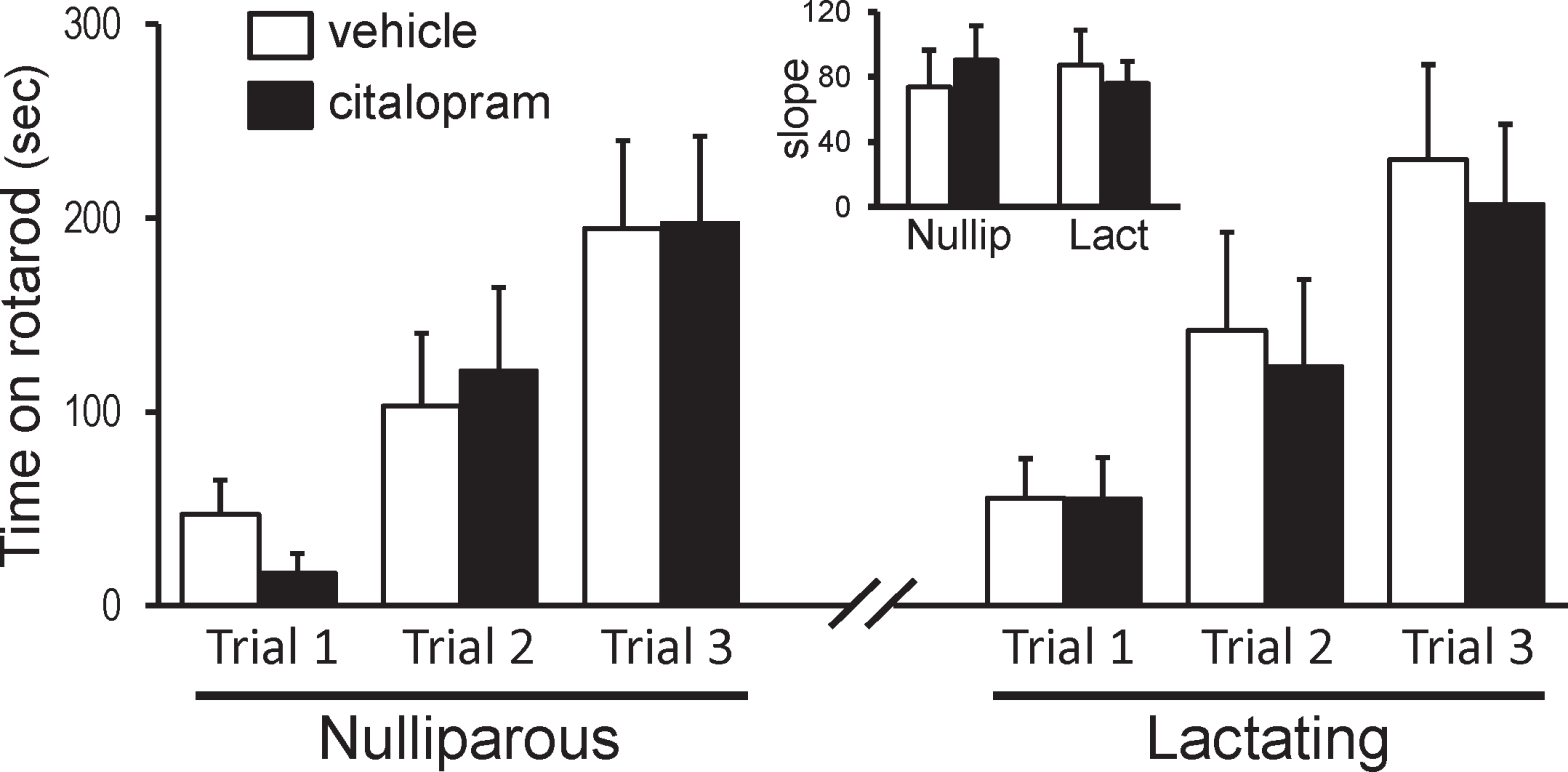
Citalopram has no effect on motor performance in either nulliparous or lactating mice. The time spend on the rotarod in each of 3 trials is shown. All animals demonstrated improved ability to stay on the rotarod with each subsequent trial. *Inset*: Means of the slopes for each animal’s performance (time over trial number, n = 8–10 per group).

SSRI treatment also had no effect on motor performance as assessed by the rotarod task in either lactating mice (day 10 postpartum) or nulliparous mice (Fig. 7). The data obtained from the rotarod task were analyzed using the Friedman test followed by a Dunn’s post-hoc analysis, which indicated that both lactating and nulliparous mice spent significantly more time on the rotarod during Trial 3 than Trial 1 for both vehicle and citalopram (5mg/kg/day) treatment groups (Lactating: q = 4.884, df = 68, *p*<0.0001; Nulliparous: q = 5.885, df = 68, *p*<0.0001, Fig. 7).

### Serum 5-HT: Measurement following FST and MBT

**Fig 8:**
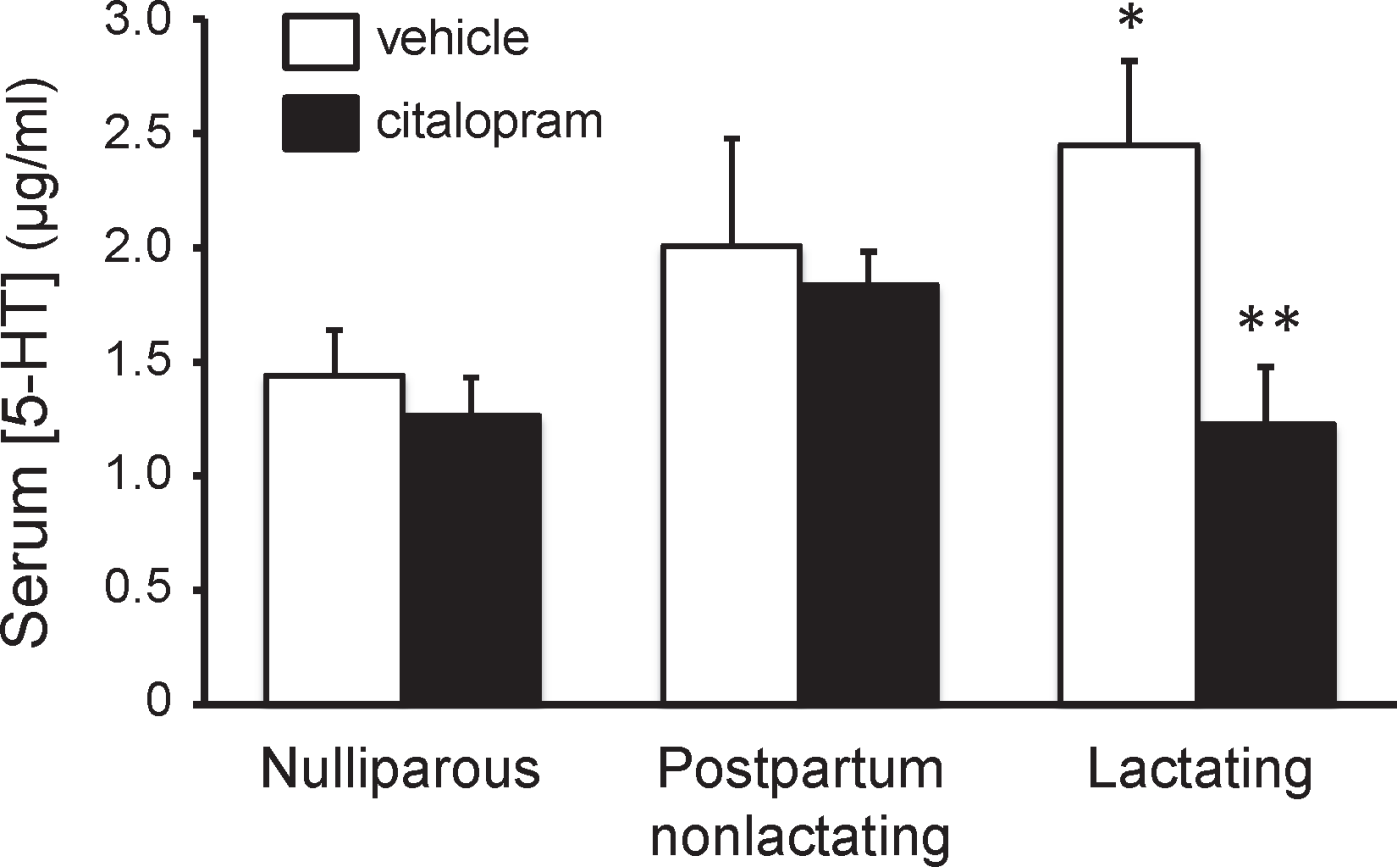
Serum 5-HT from female mice at various reproductive states, following five days of treatment with vehicle or SSRI (citalopram, 5mg/kg/day). All mice were age-matched and mated. Blood was collected from postpartum-nonlactating and lactating animals on day 10 postpartum. Trunk blood was allowed to clot overnight, releasing platelet 5-HT stores. Bonferonni post hoc test for comparison of means: *p<0.05 compared to vehicle-treated nulliparous group; **p<0.01 compared to vehicle-treated lactating group (n = 12–20 per group).

Circulating 5-HT levels were also analyzed in the mice from the behavioral experiments. Two-way ANOVA (drug treatment X lactation state) of these data determined there were significant main effects of both drug treatment [F(1,88) = 10.75; *p*<0.01] and lactation state [F(2,88) = 6.079; *p*<0.01], but the interaction between the two factors was not quite significant [F(2,88) = 2.507; *p* = 0.08]. As found in the mice used for the preliminary experiment (Fig. 3), lactating mice (vehicle-treated controls) again had significantly elevated levels of serum 5-HT (Fig. 8, [t = 2.698, df = 34, *p*<0.05]). Serum 5-HT was not significantly different between nulliparous and postpartum-nonlactating female mice. In addition, citalopram treatment significantly reduced serum 5-HT concentration in lactating mice (Fig. 8, [t = 3.618, df = 28, *p*<0.01]), but had no effect on serum 5-HT in either the nulliparous or postpartum-nonlactating groups.

### Behavioral Experiment 3: Increased Citalopram Dosage and Longer Administration

Nulliparous female mice tested at a higher dose and longer duration of citalopram (30mg/kg/day, 10 days) also showed no response to citalopram in the FST (*S2B Fig.*).

### Behavioral Experiment 4: Acute Fluoxetine and Forced Swim Test

Similarly, non-lactating females were insensitive to an acute dose of a second SSRI, fluoxetine (*S3B Fig.*), while lactating females responded to fluoxetine with a significant reduction in immobility during the FST (*S3A Fig.* [t = 3.210, df = 21, *p*<0.01]).

## Discussion

The present study produced several novel findings. First, and perhaps most significantly, lactating mice, day 10 postpartum, a time of peak lactation [27,28,29] exhibit behavioral responsiveness to SSRI treatments that have no measurable effect on non-lactating female mice. Second, lactating mice have significantly elevated serum 5-HT stores when compared to non-lactating females. Moreover, SSRI treatment significantly reduced serum 5-HT stores in lactating dams, while having no effect on those of non-lactating females. Third, immunoreactive 5-HT is significantly decreased in the dorsal raphe of lactating mice compared with nulliparous females.

Lactating mice exhibited baseline affective behavior consistent with a more positive mood compared with nonlactating females. There is literature that has reported an increase in depressive-like behavior during the postpartum period [44]. Furthermore, additional studies reported an increase in anhedonia during the postpartum period [45,46]. We did not observe an increase in depressive-like behaviors during our experiments. Our experiments were conducted in C57BL/6J mice, whereas, the aforementioned studies were conducted in Sprague-Dawley Rats, which could account for the different results. It should be noted that the majority (80–90%) of women who give birth do not experience PPD [47]. In the clinical literature the women who do decide to breastfeed often report elevations in mood [47,48]. We did not utilize a model of induced maternal depression; and our results seem to support those reported in the clinical literature. Our lactating mice also exhibited an anxiolytic phenotype (buried fewer marbles in a home cage setting) when compared to age-matched nulliparous controls. Another research group also reported a decrease in anxiety-related behaviors during the postpartum, but in contrast to our results, these anxiolytic effects were independent of lactation [49].

Lactating mice exhibited an increased responsiveness to SSRI treatments and this was true with both the sub-chronic administration of citalopram (5mg/kg/day for 5 days) and the acute administration of fluoxetine (single injection of 40mg/kg). While both block 5-HT uptake via SERT, the two SSRIs differ in structure, metabolism, and pharmacokinetics [50]. Neither SSRI altered affective behavior in nulliparous female mice. Even longer treatment with a much higher dose of citalopram (30mg/kg/day for 10 days) did not change this lack of response in the nulliparous females.

Also not responsive to SSRI treatment were female mice that had gone through a full pregnancy but had their pups removed and were not lactating. These data indicate that it is the state of lactation and/or the presence of the pups, but not parity *per se*, that underlies the change in responsiveness to SSRIs. This does not necessarily mean that pregnancy is not required to prepare the central circuitry for the alterations observed in the lactating female. The brain undergoes dramatic changes that often begin during pregnancy in preparation for parturition and lactation. The supraoptic and paraventricular nuclei, sites of oxytocin cell bodies, undergo extensive neuro-glia remodeling [51]. Also during lactation, the tuberoinfundibular dopaminergic (TIDA) neurons that normally inhibit prolactin release begin to express enkephalin, which stimulates prolactin [52]. If changes were initiated by pregnancy, the enhanced responsiveness to SSRI clearly requires the active state of lactation.

As with the measures of affective behavior, only lactating dams had a significant decrease in serum 5-HT, reflecting platelet stores, in response to SSRI treatment. In the clinical literature, various parameters of platelet 5-HT have been examined as a potential peripheral marker of SSRI efficacy. This has arisen from the search for a biochemical marker that would have greater discriminatory power than the subjective rating scales used for measuring depression. The concentration of platelet 5-HT has consistently appeared useful as a marker in data from multiple laboratories. There is a single gene for SERT and the transporter expressed in platelets is the same as that expressed in neurons [53], so SSRI treatment blocks uptake of 5-HT into platelets as well. Because platelets do not contain 5-HT biosynthetic enzymes, blocking SERT results in a gradual loss of platelet serotonin stores. It has been demonstrated by independent laboratories that the magnitude of SSRI effect on platelet 5-HT directly correlates with their efficacy in alleviating depressive symptoms as determined by standard psychometric tools such as the Hamilton Depression Rating Scale [54,55]. Correlation of SSRI-induced decrease in platelet 5-HT levels with effects on affective behavior was also seen in the present mouse experiments. Citalopram treatment significantly reduced serum 5-HT concentration only in lactating dams (Fig. 8), and the lactating mice was the only group in which citalopram (or fluoxetine) significantly altered behaviors relevant to mood as assessed by the FST and MBT (Figs. 4 and 5).

The elevated serum 5-HT storage pools in lactating dams is striking, being up to 40% greater than that in nulliparous mice. The majority of serotonin in the body is produced by the enterochromaffin cells in the mucosa of the gut [56] where it regulates peristaltic and secretory reflexes [57,58]. Serotonin produced in the gut enters the bloodstream where it is rapidly transported into platelets via SERT [53] and stored in dense-core granules [40]. 5-HT is released upon platelet activation, and functions in vasoconstriction and thrombosis. High circulating 5-HT is found in certain pathologies, such as carcinoid tumors, but lactation appears to be a unique *physiological* state in which circulating 5-HT levels are elevated. Platelet 5-HT levels do not differ between sexes or among healthy people of varying age (with the exception of newborns) [59]. Also, there is no circadian rhythm in platelet 5-HT content despite a small circadian rhythm in plasma tryptophan [60]. It is conceivable that the elevated 5-HT during lactation is traceable to 5-HT synthesis in the mammary glands [27], but it is also the case that the intestine of female mammals undergoes marked mucosal hyperplasia during lactation [61]. Thus, the elevated serum (platelet stores) 5-HT during lactation may be supplied by multiple sources.

The vast majority of cell bodies of the central serotonergic system are located within the brainstem and caudal midbrain. Of particular interest are the cell bodies contained within the DRN, as these are the origins of the major ascending serotonergic pathways to the forebrain, including numerous limbic structures. The markedly lower 5-HT immunoreactivity in the dorsal raphe nuclei (DRN) of lactating dams as compared with nulliparous controls may indicate a change in central 5-HT activity, resulting in decreased storage pools of 5-HT. However, the quantification of 5-HT staining in the DRN must be interpreted in light of other evidence. For example, the predicted effects on affective behaviors are very different in the case of reduced 5-HT synthesis or increased 5-HT transport and release. The reduced 5-HT staining in the DRN of lactating mice was particularly striking in the lateral aspects of the DRN and VLPAG. Serotonergic neurons arising from these regions project to a distributed system involved in physiological and behavioral responses associated with stress and anxiety [62]. Retrograde labeling and double-staining experiments [96] have demonstrated that the serotonergic neurons projecting to the amygdala [63] and lateral septum [64] are concentrated at the level shown in Fig. 2, A and B (Bregma -4.84). Both the amygdala and the lateral septum are key components in the brain’s emotional circuitry that modulates affective behavior and stress responses [65,66]. Increased serotonergic neurotransmission to these regions could be expected to reduce depressive-like or anxiety-related behavior.

We do not propose that 5-HT acts alone in regulating affective behavior and mood during lactation. Numerous other neurohormones and transmitters alter affective states, many of which undergo dramatic changes during pregnancy and lactation. Obvious candidates during lactation include prolactin and oxytocin, the endocrine secretions of which are required for milk production and milk let-down in the mammary gland. Receptors for both hormones are present in a number of brain regions, including those associated with affective behavior, such as the amygdala and lateral septum [67,68]. Both hormones regulate behaviors associated with reproduction, such as pair-bonding and parenting [69,70]. In addition, oxytocin has been reported to have antidepressant and anxiolytic activities [71,72]. One study found anxiolytic action of oxytocin only in pregnant and lactating rats, and not in virgin females [73]. One possible explanation for the differential responsiveness to SSRIs between non-lactating and lactating mice could be the fact that there is a higher release of oxytocin from the paraventricular (PVN) and supraotic (SON) nuclei during lactation [74]. Oxytocin has been shown to be important in pair bonding behavior [69]. Furthermore, oxytocin-labeled fibers have been found within limbic structures that could modulate mood-like behaviors [75,76]. SERT labeled fibers have been identified within the PVN and SON of non-human primates [77]. There is some evidence that SSRIs can increase the release of oxytocin in humans [78]. It could be hypothesized that the SSRIs are acting through these SERT-containing fibers, and increasing the release of oxytocin, often considered a “feel-good” modulator, in the lactating mice. This might explain the difference in responsiveness to SSRIs between non-lactating and lactating female mice.

Intracerebroventricular administration of antisense oligonucleotides against the prolactin receptor was found to increase anxiety-related behavior in lactating rats as well as impair maternal behavior [79]. 5-HT serves as a mediator in the neuroendocrine reflexes that result in the secretion of oxytocin and prolactin, including suckling-induced and stress-induced release [80,81,82]. Thus, either or both of these two hormones may be involved in the differential behavioral responsiveness to SSRIs in lactating and nonlactating mice that we report here.

In addition to being exclusive to lactating dams, the effect of SSRI treatment was specific for measures of affective and mood-related behaviors. The FST is considered a measure of behavioral coping in animals presented with an inescapable stress and has predictive validity in detecting the effects of antidepressants [36]. Treatments or psychological states that elevate mood result in decreased time of immobility. The MBT is considered a measure of anxiety-related behavior and treatments that reduce anxiety in humans result in a decrease in the number of marbles buried. Both of these behavioral tests are commonly used in the pharmaceutical industry for screening antidepressants and anxiolytics [37,38,83,84]. One potential caveat to our experiments is that we administered the SSRI via an i.p. injection instead of via oral gavage. The altered metabolism that often occurs during pregnancy and lactation could dramatically decrease the concentration of the SSRI being administered orally. There is a possibility that our route of administration may have circumvented the increase in metabolism [85]. The clinical implications of our results should be interpreted based on the administration route. Future animal studies examining the interaction between lactation and SSRI efficacy should address this difference in administration route.

5-HT is involved in numerous central functions including motor skills, learning and memory, sleep-wakefulness cycles, and others. SSRI treatment had no effect on motor peformance in the rotorod test. This test also involves some learning over the three trials and SSRI treatment did not alter the rate at which motor performance improved in any of the animals (Fig. 7). Spontaneous locomotor activity in the home cage also was unaffected by SSRI in both the nulliparous and lactating mice. The absence of a circadian rhythm in home cage activity in lactating mice was both surprising and novel (Fig. 6). Of the few studies in which circadian rhythms were assessed in lactating animals, even fewer monitored spontaneous activity. Both nocturnal rats and hamsters [86,87] have a significant reduction in the difference between the levels of spontaneous activity during the light and dark periods in lactating animals as compared with virgin or pregnant animals. Yet a significant rhythm is still present. The diurnal Nile grass rat also exhibits a circadian rhythm in activity during lactation which is even greater than that of virgin females but less than that of pregnant females [88]. Although we expected a dampening of the difference in cage activity between the light and dark periods in lactating mice, due to time spent nursing in the nest, we were surprised at the total absence of a rhythm. By day 10 of lactation, pups are more than 70% of their weight at weaning [27,28,29] and should well tolerate the dam spending time outside the nest. Whether this arhythmicity in cage activity of lactating C57BL/6J mice is characteristic of other non-maternal behaviors and/or vegetative functions (e.g. hormone rhythms) would require further investigation.

Any potential relationship between breastfeeding and maternal mood has not been definitively established. Although most studies have found a positive correlation between breastfeeding and alleviation of PPD symptoms [48,89,90], a few studies have found a higher incidence of PPD among breastfeeding women [91,92]. A recent qualitative systematic review of the literature found that most of these latter studies have methodological limits and weaknesses that equivocate conclusions about how breastfeeding influences maternal depressive symptomatology [93]. However, the authors did conclude that there is unequivocal evidence that PPD negatively influences infant-feeding outcomes.

To our knowledge, only one other study has investigated the interaction of lactation and efficacy of SSRIs [94]. Pawluski and colleagues used a model of maternal adversity to examine the effects of stress during gestation and the effects of fluoxetine on behaviors during the postpartum. In most animal models, studies on the efficacy of antidepressant treatment have been done primarily in males and, to a much lesser extent, in virgin females. The few studies that have used lactating animals have not compared their responses to those of non-lactating females. The clinical literature also has not addressed whether breastfeeding can alter responsiveness to SSRIs. Those studies that have examined SSRI use in lactating mothers have been concerned with risk assessment for negative outcomes in babies exposed to the medications during breastfeeding. Current research focuses on two aspects of antidepressant use during breastfeeding: 1) how much of the medication passes into breast milk, and 2) does the medication affect the infant?

## Conclusions

The clinical implications of these findings are significant. It is clear that untreated maternal depression has a highly negative impact on the development of the child [95], while breastfeeding is recognized as having a highly positive effect on child health and development. Yet, mothers with PPD often believe that they must make a choice between treatment and breastfeeding [97]. It may be that combined treatment *and* breastfeeding has the greatest benefit for both mother and child. Moreover, if the state of lactation does increase sensitivity to the antidepressant actions of SSRIs, then it is possible that lower doses of drug may be effective in treating PPD in many patients. What remains clear is that clinical studies on the potential interactions of lactation and SSRI efficacy are very much needed.

## Supporting Information

S1 Fig Schemas for experiments assessing behavior after treatment with citalopram or fluoxetine. ***A***: FST Pilot Experiment: the effectiveness of subchronic treatment (5 days) with citalopram (5mg/kg/day) and determined replicability of previous published results. ***B***: Behavioral Experiment 3: the effects of an increased dose (30mg/kg/day) and duration (10 days) of citalopram in nulliparous mice during the FST. ***C***: Behavioral Experiment 4: 1) *Left panel*: lactating mice (day 10 postpartum) were treated with a single dose of fluoxetine (10mg/kg or 40mg/kg), or vehicle 30 minutes prior to the FST. 2) *Right panel*: nulliparous mice were given a single injection of fluoxetine (40mg/kg) or vehicle 30 minutes prior to the FST.
(TIFF)

S2 Fig Citalopram treatment reduces total immobility during FST in male C57Bl6 mice, but not in nulliparous female mice. ***A*.** Male mice respond to regimen of citalopram treatment that has no effect on nulliparous or postpartum-nonlactating female mice (5 days @ 5mg/kg/day, i.p.; see Fig. 4; n = 15 per group). ***B***. Nulliparous female mice do not respond to increased dose and duration of citalopram. Mice received daily injections of vehicle or citalopram (30mg/kg/day, i.p.) for 10 days (n = 6 per group). Student’s t-test for comparison of the means: **p<0.05 compared with vehicle-treated controls.
(TIFF)

S3 Fig Lactating mice, but not nulliparous female mice, respond to acute fluoxetine treatment in the FST. All mice received a single injection of vehicle or fluoxetine (Flx; 10 or 40 mg/kg) and subjected to FST 90 minutes later. **A.** Acute SSRI reduced total immobility time in the FST (n = 9–14 per group). **B**. Acute SSRI treatment (fluoxetine, 40mg/kg) had no effect on total immobility time nulliparous female C57/Bl6 mice (n = 11 per group). **p<0.01 compared vehicle-treated controls.
(TIFF)

S4 Fig Separation from pups increases marble-burying activity of lactating female mice. The number of marbles buried by lactating mice with pups or separated from their pups was measured either in their home cage or a novel cage. Bonferonni post hoc for comparison of means: ***p<0.001 vs. respective “with pups” group (n = 5–8 per group).
(TIFF)
