# Supplementary figures and images for: Enhanced Responsiveness to Selective Serotonin Reuptake Inhibitors during Lactation
Source: PLoS One. 2015 Feb 17;10(2):e0117339. doi: 10.1371/journal.pone.0117339 (PMC4331562; doi:10.1371/journal.pone.0117339)

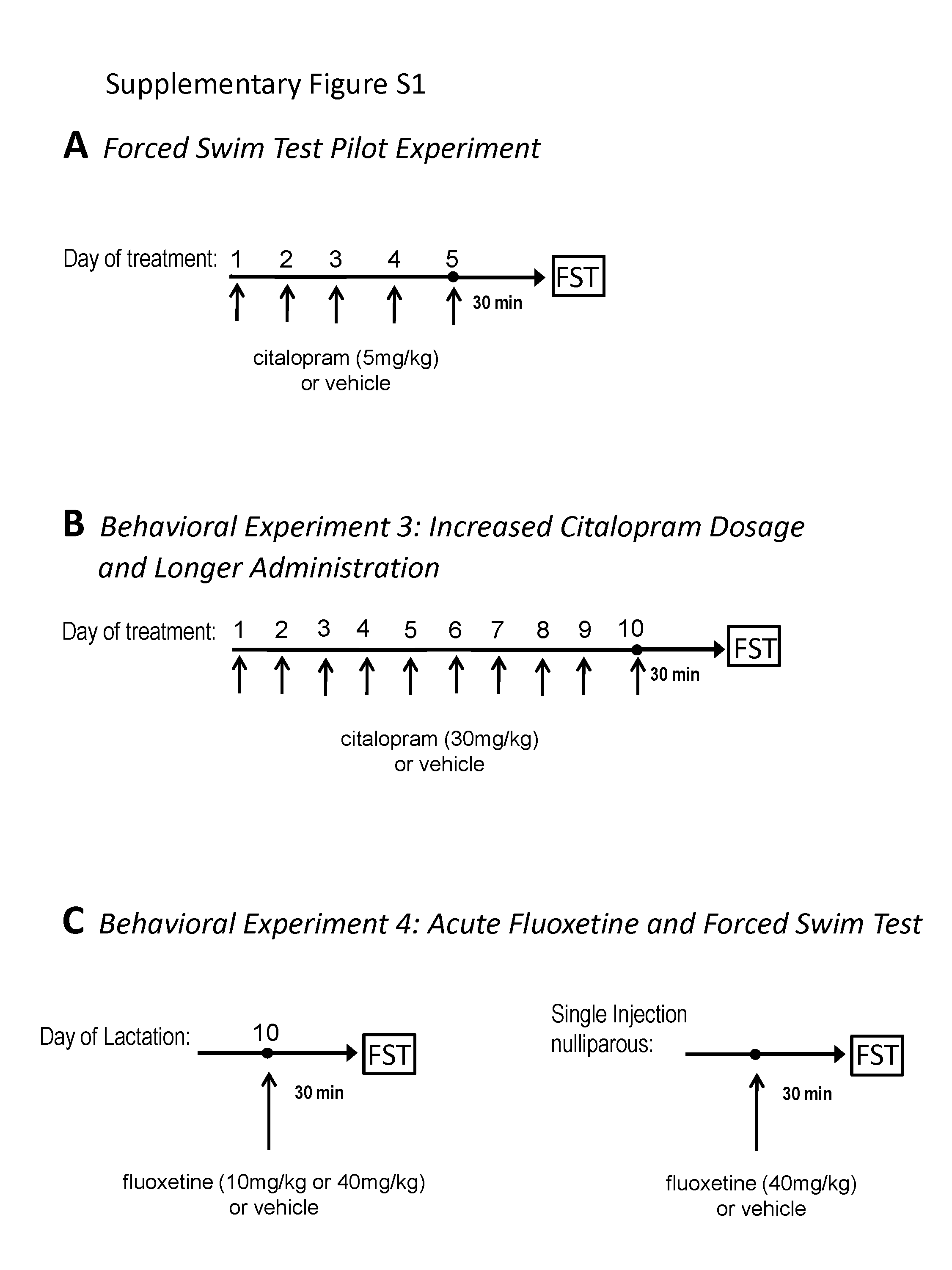

Supplement: S1 Fig — A: FST Pilot Experiment: the effectiveness of subchronic treatment (5 days) with citalopram (5mg/kg/day) and determined replicability of previous published results. B: Behavioral Experiment 3: the effects of an increased dose (30mg/kg/day) and duration (10 days) of citalopram in nulliparous mice during the FST. C: Behavioral Experiment 4: 1) Left panel: lactating mice (day 10 postpartum) were treated with a single dose of fluoxetine (10mg/kg or 40mg/kg), or vehicle 30 minutes prior to the FST. 2) Right panel: nulliparous mice were given a single injection of fluoxetine (40mg/kg) or vehicle 30 minutes prior to the FST. (TIFF) [file pone.0117339.s001.tiff]

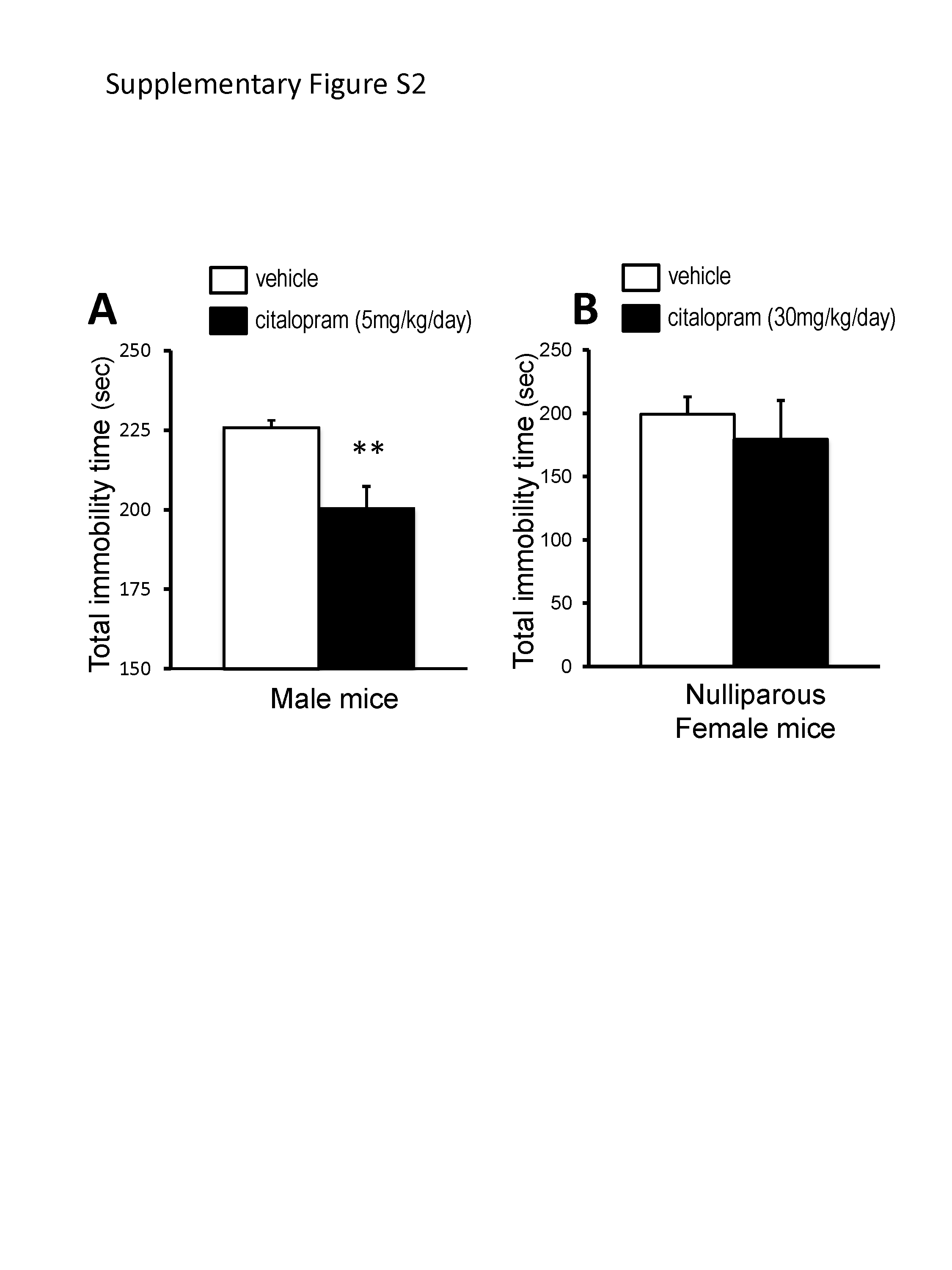

Supplement: S2 Fig — A. Male mice respond to regimen of citalopram treatment that has no effect on nulliparous or postpartum-nonlactating female mice (5 days @ 5mg/kg/day, i.p.; see Fig. 4; n = 15 per group). B. Nulliparous female mice do not respond to increased dose and duration of citalopram. Mice received daily injections of vehicle or citalopram (30mg/kg/day, i.p.) for 10 days (n = 6 per group). Student’s t-test for comparison of the means: **p<0.05 compared with vehicle-treated controls. (TIFF) [file pone.0117339.s002.tiff]

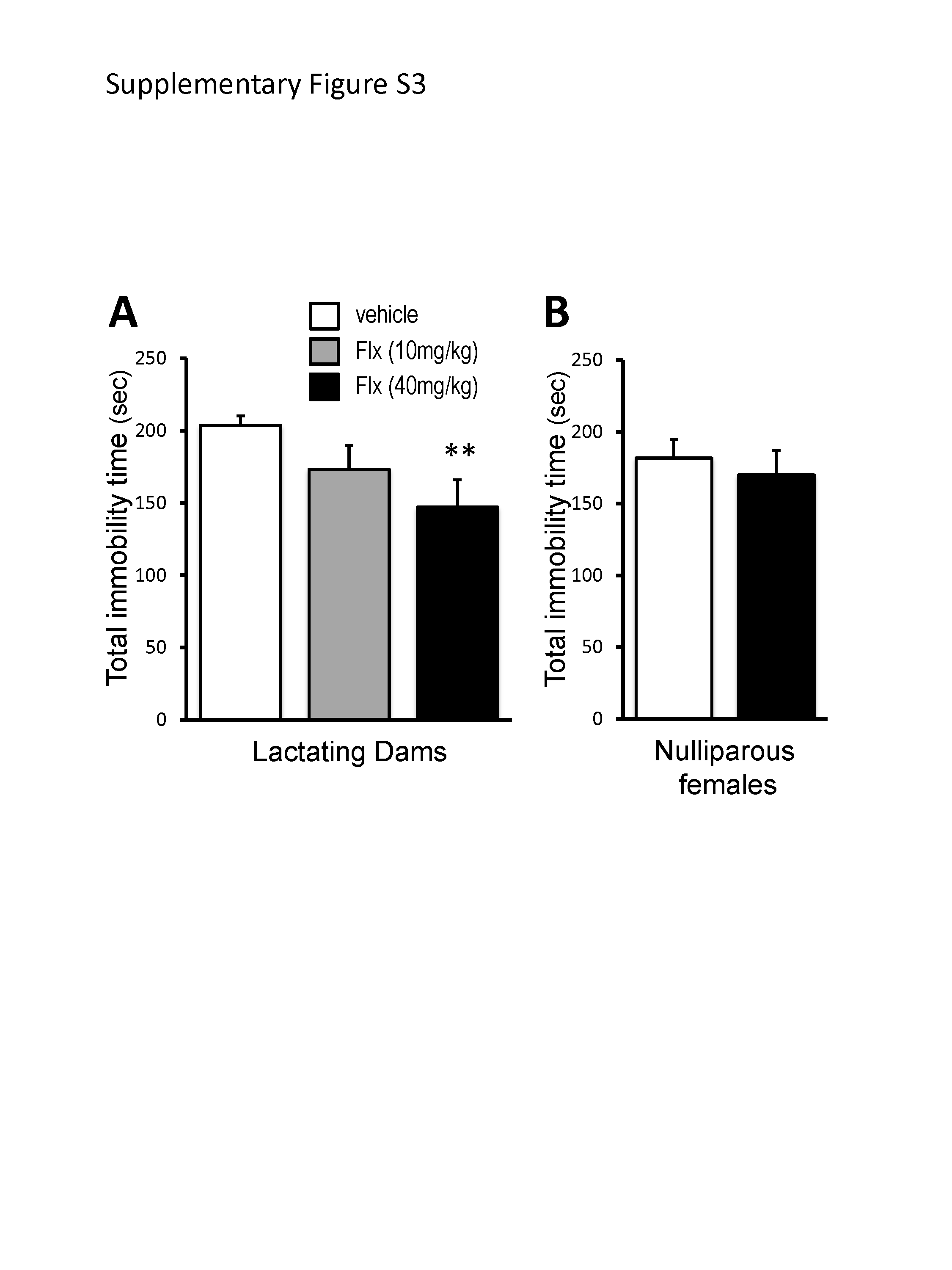

Supplement: S3 Fig — All mice received a single injection of vehicle or fluoxetine (Flx; 10 or 40 mg/kg) and subjected to FST 90 minutes later. A. Acute SSRI reduced total immobility time in the FST (n = 9–14 per group). B. Acute SSRI treatment (fluoxetine, 40mg/kg) had no effect on total immobility time nulliparous female C57/Bl6 mice (n = 11 per group). **p<0.01 compared vehicle-treated controls. (TIFF) [file pone.0117339.s003.tiff]

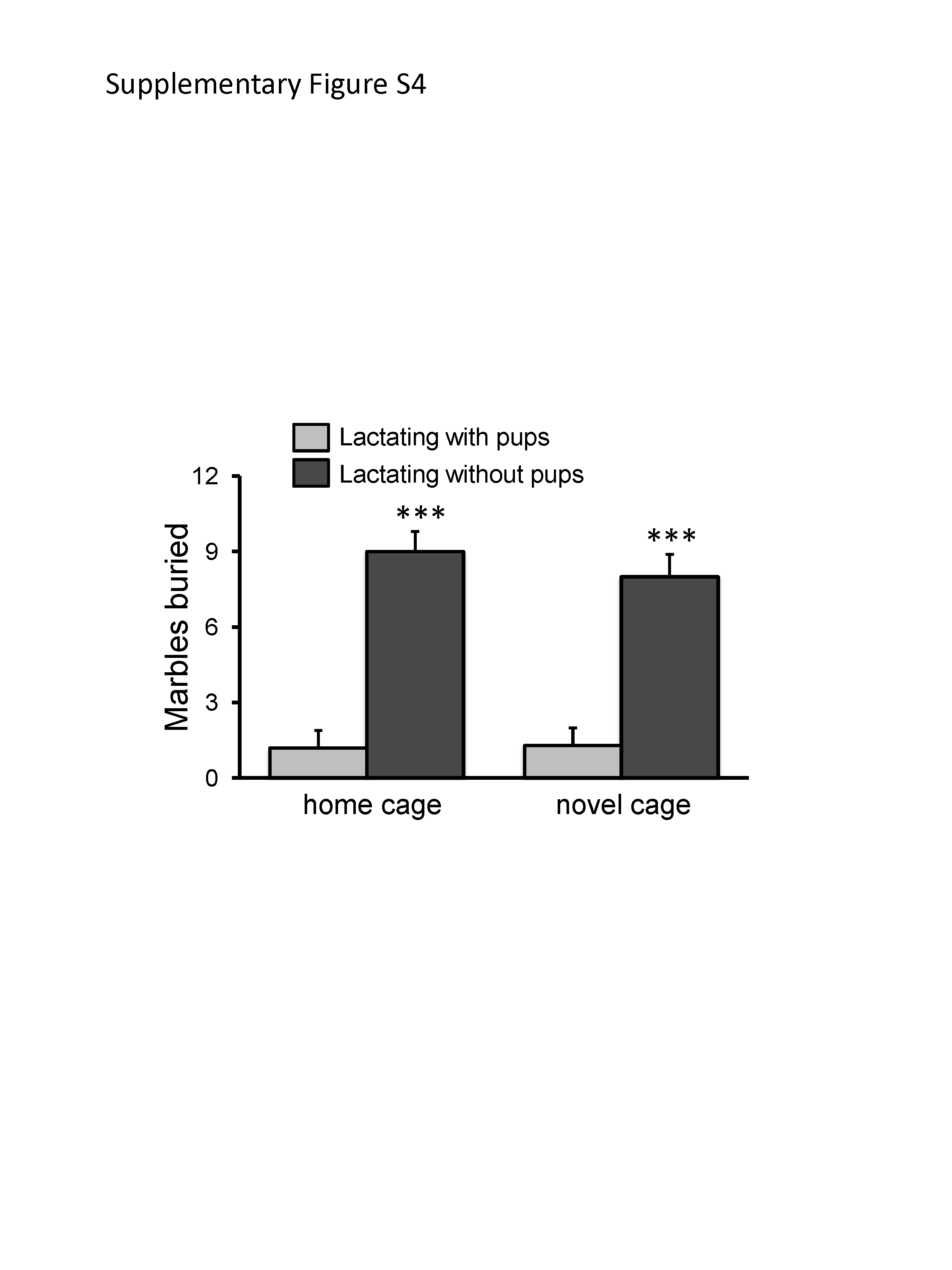

Supplement: S4 Fig — The number of marbles buried by lactating mice with pups or separated from their pups was measured either in their home cage or a novel cage. Bonferonni post hoc for comparison of means: ***p<0.001 vs. respective “with pups” group (n = 5–8 per group). (TIFF) [file pone.0117339.s004.tiff]
